# Generation of composite *Persea americana* (Mill.) (avocado) plants: A proof-of-concept-study

**DOI:** 10.1371/journal.pone.0185896

**Published:** 2017-10-20

**Authors:** S. Ashok Prabhu, Buyani Ndlovu, Juanita Engelbrecht, Noëlani van den Berg

**Affiliations:** 1 Forestry and Agricultural Biotechnology Institute (FABI), University of Pretoria, Pretoria, South Africa; 2 Department of Microbiology and Plant Pathology, University of Pretoria, Pretoria, South Africa; Karnatak University, INDIA

## Abstract

Avocado (*Persea americana* (Mill.)), an important commercial fruit, is severely affected by Phytophthora Root Rot in areas where the pathogen is prevalent. However, advances in molecular research are hindered by the lack of a high-throughput transient transformation system in this non-model plant. In this study, a proof-of-concept is demonstrated by the successful application of *Agrobacterium rhizogenes*-mediated plant transformation to produce composite avocado plants. Two *ex vitro* strategies were assessed on two avocado genotypes (Itzamna and A0.74): In the first approach, 8-week-old etiolated seedlings were scarred with a sterile hacksaw blade at the base of the shoot, and in the second, inch-long incisions were made at the base of the shoot (20-week-old non-etiolated plants) with a sterile blade to remove the cortical tissue. The scarred/wounded shoot surfaces were treated with *A*. *rhizogenes* strains (K599 or ARqua1) transformed with or without binary plant transformation vectors pRedRootII (DsRed1 marker), pBYR2e1-GFP (GFP- green fluorescence protein marker) or pBINUbiGUSint (GUS- beta-glucuronidase marker) with and without rooting hormone (Dip 'N' Grow) application. The treated shoot regions were air-layered with sterile moist cocopeat to induce root formation. Results showed that hormone application significantly increased root induction, while *Agrobacterium*-only treatments resulted in very few roots. Combination treatments of hormone+*Agrobacterium* (-/+ plasmids) showed no significant difference. Only the ARqua1(+plasmid):A0.74 combination resulted in root transformants, with hormone+ARqua1(+pBINUbiGUSint) being the most effective treatment with ~17 and 25% composite plants resulting from strategy-1 and strategy-2, respectively. GUS- and GFP-expressing roots accounted for less than 4 and ~11%, respectively, of the total roots/treatment/avocado genotype. The average number of transgenic roots on the composite plants was less than one per plant in all treatments. PCR and Southern analysis further confirmed the transgenic nature of the roots expressing the screenable marker genes. Transgenic roots showed hyper-branching compared to the wild-type roots but this had no impact on *Phytophthora cinnamomi* infection. There was no difference in pathogen load 7-days-post inoculation between transformed and control roots. Strategy-2 involving A0.74:ARqua1 combination was the best *ex vitro* approach in producing composite avocado plants. The approach followed in this proof-of-concept study needs further optimisation involving multiple avocado genotypes and *A*. *rhizogenes* strains to achieve enhanced root transformation efficiencies, which would then serve as an effective high-throughput tool in the functional screening of host and pathogen genes to improve our understanding of the avocado-*P*. *cinnamomi* interaction.

## Introduction

Avocado (*Persea americana* (Mill.)) is a major tropical fruit along with mango, pineapple and papaya, accounting for approximately two-thirds of the world tropical fruit production. In 2014, global avocado production was estimated to be 5.02 million tons of which South Africa contributed 107176 tons [1]. Avocado production is of economic importance to South Africa, as 65845 tons of avocados with a total value of R978 million was exported in 2014 [2]. In addition to consumption, avocado is also processed for oil and guacamole and used in the cosmetics industry due to high nutritional content. The most important biotic constraint in avocado production in South Africa is Phytophthora Root Rot (PRR) caused by the oomycete *Phytophthora cinnamomi* Rands, a pathogen with a broad host range of over 3500 plant species and found in all major avocado producing areas in the world [3,4]. PRR affects trees of all ages, including those in nurseries and acts through the destruction of the feeder roots [5,6]. The incidence of PRR across the world has been reported to vary, but can be as high as 90%, leading to wipe out of orchards or significantly limiting fruit production [7]. At present, PRR can only be managed by an integrated approach that includes the use of tolerant rootstocks and orchard management such as mulching and controlled irrigation [7]. In addition, chemical control in the form of phosphite spray or trunk injections has extensively been used to control PRR [8].

Avocado is heterogeneous with a prolonged juvenile period and the lack of a published genome sequence has hindered the application of conventional breeding of avocado for PRR resistance [9]. Hence, selection by screening thousands of seedlings for tolerance to *P*. *cinnamomi* has been the only way to identify promising plant material. Less than 1% of the progeny of a PRR-resistant parent has been shown to inherit the resistance trait, therefore, clonal propagation of tolerant rootstocks remains the only choice for sustainable production [10].

Functional analysis of *P*. *cinnamomi* pathogenicity factors and avocado defense machinery is an important research objective, in order to identify potential molecular targets for positive exploitation in marker-assisted avocado rootstock selection and improvement programmes. However, the luxury of genetic resources available in model plants is lacking in avocado. The application of reverse genetic tools such as Targeting Induced Local Lesions in Genomes and genome editing for high-throughput functional studies in the present system are not feasible, due to the inherent problems associated with the tools, as well as the host, as mentioned earlier. In addition, a successful transformation protocol for the pathogen—*P*. *cinnamomi* has to date not been published.

Plant transformation tools are critical in the functional analysis of genes. Simple transformation tools such as a floral-dip method, vacuum infiltration and syringe infiltration of *Agrobacterium tumefaciens*, commonly employed in model plants such as *Arabidopsis thaliana* and *Nicotiana benthamiana* are not easily adapted to non-model woody plants such as avocado. Though *A*. *tumefaciens*-based plant transformation and regeneration protocols have been previously reported in avocado [9,11–14], it is not amenable to high-throughput whole plant transient transformation studies for the systemic dissection of the defense pathways. Further, the available transformation protocols employ plant tissue culture which requires sterile conditions necessitating specialized infrastructure and training [15].

*A*. *rhizogenes*, a Gram-negative soil bacterium from the family *Rhizobiaceae* was identified as the reason behind the hairy-root disease [16]. Since then, the bacterium has been shown to induce adventitious roots in over 450 plant species [17]. The hairy-roots are characterized by plagiotropism, high lateral branching and their ability to grow and proliferate in hormone-free media. The manifestations have been shown to be due to the transfer of Ri (root inducing)-plasmid T-DNA containing the *rol* (root loci) genes into the plant cells. A combination of *rol* genes A, B and C was shown to be sufficient for root induction in plants [18]. Initially, *A*. *rhizogenes* was exploited in secondary metabolite production via the establishment of hairy-root cultures. As the bacterium was also shown to be useful in the transfer of foreign genes into plants, it has been employed in gene and promoter analysis, generation of stable transgenic plants, root biology and root-biotic interaction studies [17,19]. The major limitations of the hairy root cultures were their restriction to tissue culture bottles and lack of a whole plant system for studying the effect of abiotic and biotic interactions at the systemic level. To partially mitigate the above-mentioned drawbacks Hansen and his co-workers came up with the concept of *A*. *rhizogenes*-mediated ‘composite plants’ a chimera of wild-type shoots bearing a mix of transgenic and non-transgenic roots [20]. However, it was still an *in vitro* approach. A novel *ex vitro* method was devised by [21] to produce composite plants which provided a simple, cost-effective whole-plant system for the functional analysis under non-axenic conditions. The tool has been demonstrated to be applicable in a number of transformation recalcitrant dicotyledonous plants. The system is amenable to not just gene overexpression studies but also to RNAi-based gene silencing studies facilitating host-induced gene silencing. With this background the present study aimed to generate a whole plant transformation tool in avocado using an *ex vitro* approach for potential down-stream application in the functional genetic dissection of the avocado-PRR interaction in future. A proof-of-concept is demonstrated by the successful application of *A*. *rhizogenes*-mediated plant transformation to produce composite avocado plants.

## Materials and methods

### Plant material and *P*. *cinnamomi* isolate

Avocado seeds of A0.74 and Itzamna were obtained from Westfalia Technological Services, Tzaneen, Limpopo, South Africa. *P*. *cinnamomi* isolate GKB4 used in avocado infection experiments was obtained from the culture collection of the Avocado Research Programme, Forestry and Agricultural Biotechnology Institute (FABI), University of Pretoria, Pretoria, South Africa.

### Bacterial strains and plasmid vectors

#### Bacterial strains

*A*. *rhizogenes* strains- K599 [22] and ARqua1 were kindly provided by Prof. Bettina Hause (Department of Cell and Metabolic Biology, Leibniz Institute of Plant Biochemistry, Weinberg 3, 06120 Halle (Saale), Germany).

#### Plasmid vectors

pRedRootII containing a DsRED1 marker [23] (DsRed1- red fluorescence protein) (kindly provided by Dr. Erik Limpens, Department of Plant Sciences, Laboratory of Molecular Biology, Wageningen University & Research, Droevendaalsesteeg 1, Wageningen 6708 PB, The Netherlands), pBYR2e1-GFP containing a GFP (- green fluorescence protein) marker (kindly provided by Dr. Hugh Mason, Biodesign Institute, School of Life Sciences, Arizona State University, Tempe, Arizona, USA) and pBINUbiGUSint containing a GUS (- β-glucuronidase) marker (a kind gift from Prof. Fernando Pliego Alfaro, Department of Plant Biology, University of Malaga, Spain).

### Generation of composite avocado plants

#### Preparation of *A*. *rhizogenes* for avocado transformation

The *A*. *rhizogenes* strains- K599 and ARqua1 were electroporated individually with the plasmid vectors pRedRootII, pBYR2e1-GFP and pBINUbiGUSint using the Eppendorf Eporator^®^ (Eppendorf, Hamburg, Germany) according to the manufacturer’s instructions. Single bacterial colonies containing the individual plasmids were grown separately in 1 L Erlenmeyer flasks containing 200 mL Luria Bertani (LB) broth amended with appropriate antibiotic combinations: 50 mg/L kanamycin for the vectors, and in addition, 100 mg/L streptomycin for ARqua1 strain. Flasks were incubated at 28°C, 180 rpm overnight. The bacterial cells were pelleted by centrifugation at 3000 rpm for 10 min at 4°C. Pellets were washed thrice with 1/4x strength Murashige and Skoog Basal Media (MSBM), pH 5.2 to remove traces of the antibiotics, resuspended in the same medium containing 100 μM acetosyringone to A_600nm_ 1.0 and left undisturbed in the dark for 3 h at room temperature.

#### Growth of avocado plants for agroinfection

Avocado seeds were surface sterilized by immersion in a solution containing 0.5% (v/v) sodium hypochlorite, 10 drops/L Tween 20, 0.01% (w/v) ascorbic acid and 0.2% (w/v) citric acid for 15 min. The seeds were subsequently rinsed with sterile distilled water thrice for 15 min each. Seeds were planted in 8 L bags filled with sterile soil:bark (1:1) mixture. One set of the seeds were incubated in the dark for 8 weeks at 25°C to induce etiolation. A second set were grown for 20 weeks under 16 h light/8 h dark in a phytotron at 25°C.

#### Plant treatments

**Fig 1:**
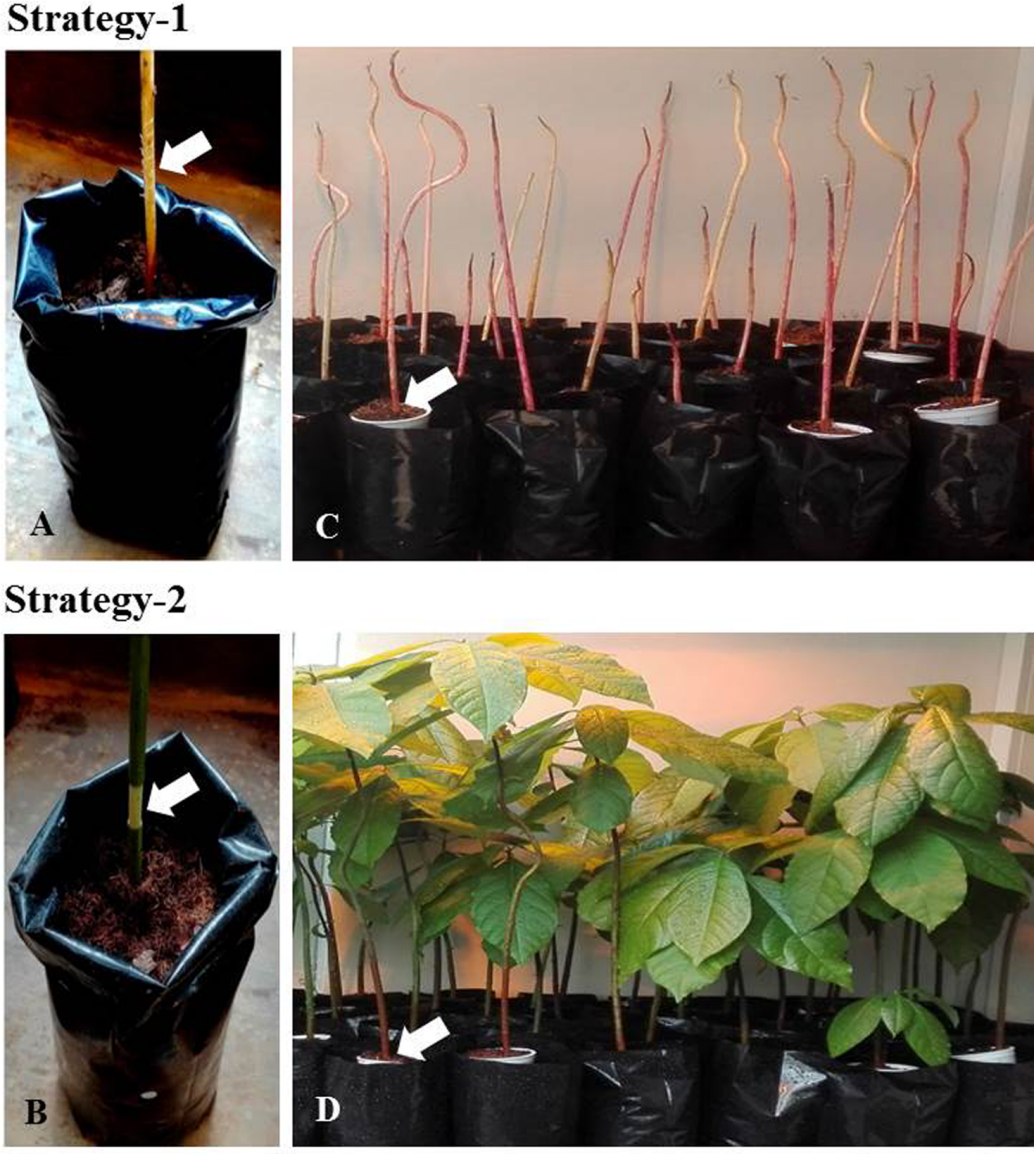
Generation of *ex vitro* composite avocado plants by air-layering. Strategy-1: 8-week-old etiolated seedlings scarred six times with a sterile hacksaw blade at the base of the shoot on opposite surfaces **(A)**; Strategy-2: One-inch-long incisions were made at the shoot base of 20-week-old plants with a sterile surgical blade to remove the cortical tissue **(B)**; Scarred/wounded shoot surfaces covered with a 175 mL foam cup filled with sterile moist cocopeat post various treatments as listed in **Table 1 (C, D)**.

**Strategy-1:** The 8-week-old etiolated seedlings with uniform stem thickness were scarred six times with a sterile hacksaw blade (Harden blades 12 x ½” x 24T) at the base of the shoot on opposite surfaces **(Fig 1A)**.

**Strategy-2:** One-inch-long incisions were made at the shoot base of 20-week-old plants (with uniform stem thickness) grown under 16 h light/8 h dark conditions with a sterile surgical blade to remove the cortical tissue **(Fig 1B)**.

The scarred/wounded shoot surfaces were immediately subjected to treatments as detailed in **Table 1**, using separate paint brushes. The treated shoot regions were covered with a 175 mL foam cup filled with sterile moist cocopeat **(Fig 1C and 1D)**. Plants were maintained under 16 h light/8 h dark in a phytotron at 25°C and the root induction was monitored weekly taking care not to disturb the rooting process. In both approaches three biological replicates of three plants each were employed.

**Table 1:** Treatments assessed in the generation of *ex vitro* composite avocado plants.

Treatment ID	Shoot Applications
DGMS	1% Dip-Gel™ in 1/4x MSBM, pH 5.2
H	Dip’N Grow liquid rooting concentrate diluted 1:5 with 1/4x MSBM, pH 5.2
ARK	DGMS + *Agrobacterium rhizogenes* K599
ARQ	DGMS + *A*. *rhizogenes* ARqua1
H + ARK	H application followed by ARK treatment
H + ARQ	H application followed by ARQ treatment
ARK-RRII	DGMS + *A*. *rhizogenes* K599 (+pRedRootII)
ARK-GFP	DGMS + *A*. *rhizogenes* K599 (+pBYR2e1-GFP)
ARK-GUS	DGMS + *A*. *rhizogenes* K599 (+pBINUbiGUSint)
H + ARK-RRII	H application followed by ARK-RRII treatment
H + ARK-GFP	H application followed by ARK-GFP treatment
H + ARK-GUS	H application followed by ARK-GUS treatment
ARQ-RRII	DGMS + *A*. *rhizogenes* ARqua1 (+pRedRootII)
ARQ-GFP	DGMS + *A*. *rhizogenes* ARqua1 (+pBYR2e1-GFP)
ARQ-GUS	DGMS + *A*. *rhizogenes* ARqua1 (+pBINUbiGUSint)
H + ARQ-RRII	H application followed by ARQ-RRII treatment
H + ARQ-GFP	H application followed by ARQ-GFP treatment
H + ARQ-GUS	H application followed by ARQ-GUS treatment

### Evaluation of plant transformation

#### Detection of GFP and DsRED1 fluorescence in roots

Six weeks post-treatment the plant roots were screened for the expression of GFP and DsRED1 using DFP-1^™^ Dual Fluorescent Protein Flashlight (NIGHTSEA, Bedford, MA, USA). GFP and DsRED1 were visualised with royal blue (excitation 440–460 nm, emission 500 nm long-pass) and green (excitation 510–540 nm, emission 600 nm long-pass) flashlights, respectively. Adobe Photoshop 5.5 was used to process the images.

#### Confocal fluorescence microscopy of roots

Roots were also observed under the confocal laser scanning fluorescence microscope- Zeiss LSM 510 META (Carl-Zeiss, Jena, Germany). For GFP/DsRED1 imaging laser excitation of 488 nm/505-550 nm emission band-pass and laser excitation of 543 nm/560 nm emission long-pass were used, respectively. The images were captured using the AxioCam (Carl-Zeiss) attached to the microscope and processed as described above.

#### GUS activity staining of roots

The indigogenic GUS activity staining was carried out according to the method of [24] with modifications. Putative transformed root sections were incubated in 100 mM sodium phosphate buffer (pH 7.0) containing 10 mM EDTA (pH 8.0), 0.1% (v/v) Triton-X100, 1 mM X-Gluc (5-bromo-4-chloro-3-indolyl-beta-D-glucuronic acid, cyclo- hexylammonium salt), 1 mM K_4_Fe(CN)_6_ and 1 mM K_3_Fe(CN)_6_ for 24 h at 37°C, in the dark. The roots were subsequently washed with distilled water and observed under SteREO Discovery.V12 stereomicroscope (Carl-Zeiss) fitted with AxioCam ICc5 (Carl-Zeiss) to capture images.

### Molecular confirmation of transgenic roots

#### Treatment of roots with antibiotics to eliminate *Agrobacterium*

Individual putative transformed roots were treated with 1/4x MSBM, pH 5.2 fortified with 200 mg/L cefotaxime and 200 mg/L carbenicillin for 2 h at 200 rpm and 25°C. Subsequently, root sections were sub-cultured for 4 weeks, at weekly intervals, in 1/4x MSBM, pH 5.2 with 100 mg/L cefotaxime and 100 mg/L carbenicillin. Further, the root sections were sub-cultured for 1 week in 1/4x MSBM, pH 5.2 without antibiotics to assess the presence of persistent bacteria.

#### Root DNA isolation

Total DNA was isolated from the homogenized individual roots (500 mg) with extraction buffer containing 100 mM Tris-HCl pH 8.0, 25 mM EDTA, 2 M NaCl, 2% (w/v) CTAB (Cetyltrimethylammonium bromide), 2% (w/v) polyvinylpyrrolidone K30, 500 mg/L spermidine, 2% (v/v) 2-mercaptoethanol and 5% (w/v) polyvinylpolypyrrolidone [25]. The extractions were incubated at 65°C for 30 min, and chloroform extracted at a 1:1 ratio. DNA was precipitated from the supernatant with isopropanol and treated with 2 U RNase A (Qiagen, Valencia, CA, USA) followed by additional chloroform extraction and precipitation steps as mentioned above. DNA was quantified with a NanoDrop® ND-1000 spectrophotometer (Nanodrop Technologies Inc., Montchanin, DE, USA) and stored at –20°C until further use.

#### PCR analysis

PCR analysis of the root DNA for *dsred1*, *gus* (*uidA*), *gfp*, *rolB* and *virC* genes was carried out to assess the successful transformation of the roots. PCR reactions were carried out in 20 μL reaction volumes containing 2.0 μl of 10× PCR reaction buffer, 200 μM of each dNTP, 30 ng DNA as template and 200 nM each of the forward and reverse primers specific for *dsred1*, *gus*, *gfp*, *rolB* and *virC* **(Table 2)**. The amplification was carried out using 1 U FastStart™ Taq DNA Polymerase (Roche Diagnostics GmBH) under the following conditions: initial denaturation at 94°C for 4 min followed by 30 cycles of denaturation at 94°C for 45 s, annealing at 60°C for 1 min and extension at 72°C for 1 min with a final extension of 7 min at 72°C. The amplified products were visualized on a 1.5% agarose gel stained with GelRed (Biotium Inc., Hayward, CA, USA) using the Gel Doc^™^ EZ Imager (Gel Doc™ EZ Gel Documentation System, Bio-Rad Laboratories, Hercules, CA, USA). Amplifications were performed in a Veriti™ 96-Well Thermocycler (Applied Biosystems, Singapore).

**Table 2:** Primers used in the study.

Gene	Primer ID	Nucleotide Sequence (5’-3’)	Expected Amplicon Size (in bp)
**PCR confirmation of root transformants**			
*dsred1*	dsred1F	GAGCGCGTGATGAACTTCGAG	319
	dsred1R	CCAGCTTGGAGTCCACGTAG	
*gus*	gusF	ACTGAACTGGCAGACTATCC	588
	gusR	TAAGGGTAATGCGAGGTACG	
*gfp*	gfpF	AAGGGCGAGGAGCTGTTCAC	344
	gfpR	TCGCCCTCGAACTTCACCTC	
*rolB*	rolB-F	TCTCACTCCAGCATGGAGCC	616
	rolB-R	TATCCCGAGGGCATTTTTGGTG	
*virC*	virCF	ATCATTTGTAGCGACT	730
	virCR	AGCTCAAACCTGCTTC	
**Pathogen load determination in roots**			
*LPV3*	LPV3-for	GTGCAGACTGTCGATGTG	450
	LPV3-rev	GAACCACAACAGGCACGT	
	LPV3N-for	GTCACGACCATGTTGTTG	77
	LPV3N-rev	GAGGTGAAGGCTGTTGAG	
*Actin*	Actin-for	GTATTCATTCACCACTACTG	77
	Actin-rev	AGTCAAGAGCCACATAAG	

#### Southern blot analysis

DNA (5 μg each) isolated from two individual A0.74 roots each positive for GFP and GUS expression was digested with *Hin*dIII (Thermo Fisher Scientific) individually and electrophoretically separated on a 0.8% agarose gel. The DNA was transferred to a positively charged nylon membrane (Roche Dignostics GmbH) as per the manufacturer’s instructions. As hybridization probes, *Hind*III restriction fragments of pBYR2e1-GFP and pBINUbiGUSint plasmids were random-prime labelled separately using a DIG High Prime DNA Labeling and Detection Starter kit II (Roche Dignostics GmbH). Pre-hybridization, hybridization, and chemiluminescent detection were performed as described previously [26]. Hybridization was carried out at 60°C.

### Comparison of the infection of transgenic and wild-type avocado roots by *P*. *cinnamomi*

#### Preparation of *P*. *cinnamomi* zoospore suspension

*P*. *cinnamomi* isolate GKB4 was grown on 5% V8 agar plates [27] for 4 days. Mycelial blocks (5 x 5 mm) from the actively growing regions of the plates were sub-cultured on petri plates (10 blocks per petri plate) containing 25 mL of 2% V8 broth for 3 days at 25°C. The broth was discarded and the mycelia were rinsed thrice with sterile distilled water. To induce sporangia formation plates were further incubated for 3 days at 25°C under ultraviolet light in 25 mL of Whatman 1-mm-filtered stream water per plate. The plates were cold shocked by incubating at 4°C for 1 h. Once adequate mature sporangia were observed plates were incubated at 25°C for 1 h to stimulate zoospore release. The zoospore suspension was decanted, diluted to 5x10^4^ zoospores/mL and used for root inoculations.

#### Root inoculation and harvesting

Avocado A0.74 roots expressing GFP and non-transformed A0.74 roots, three each, were surface serilized in a solution containing 0.5% (v/v) sodium hypochlorite, 10 drops/L Tween 20, 0.01% (w/v) ascorbic acid and 0.2% (w/v) citric acid for 10 min. The roots were subsequently rinsed with sterile distilled water thrice for 10 min each. Roots were inoculated by submerging them in a petri plate containing 25 mL zoospore suspension (5×10^4^ zoospores/mL). Roots mock-inoculated by submersion in sterile water served as the negative control. Roots were incubated for 3 h at 25°C followed by incubation in tissue culture bottles containing 25 mL sterile 1/4x MSBM, pH 5.8 for 7 days at 25°C, 30 rpm. Roots were washed in sterile distilled water, observed for root rot symptoms and photographed with the DSC-W320-14.1MP-Digital-Camera (Sony, Tokyo, Japan). Adobe Photoshop 5.5 was used in the electronic processing of the images. Root material was then harvested, snap-frozen in liquid nitrogen and stored at -80°C until DNA extraction.

#### DNA extractions from mycelia and roots

DNA from *P*. *cinnamomi* mycelia was isolated by using PrepMan Ultra Reagent (Applied Biosystems, Carlsbad, CA, USA). Mycelia was added (100 mg) to a 1.5-mL Eppendorf tube containing 100 μl PrepMan Ultra Sample Preparation Reagent (Thermo Fisher Scientific) and DNA was extracted according to the manufacturer’s instructions. DNA from infected and uninfected avocado root material (50 mg of the root tissue from the root tip) was extracted in the same manner. DNA concentrations were determined using the NanoDrop® ND-1000 spectrophotometer.

#### Determination of pathogen load

The pathogen load was determined according to the method developed by [27]. Standard curves for avocado (100 ng to 160 pg uninfected A0.74 root DNA) and *P*. *cinnamomi* (20 ng to 32 pg mycelial DNA) were prepared separately using *Actin* (for plants) and *LPV3N* (for pathogen) primer pairs, respectively. The amounts of plant and *P*. *cinnamomi* DNA present within samples were quantified using one-step and nested qPCR approach, respectively. The amounts of plant and pathogen DNA in the samples were deduced from the standard curves.

***LPV3* outer PCR.** PCR reactions were carried out in 20 μL reaction volumes with 50 ng root DNA as template (or different concentrations of mycelial DNA in case of standard curve preparation) with primers specific for the *LPV3* gene [28] **(Table 2)**. The reaction mix contained 2.0 μl of 10× PCR reaction buffer, 200 μM of each dNTP, 200 nM each of forward and reverse gene specific primers and 1 U FastStart™ Taq DNA Polymerase (Roche Dignostics GmbH). The following cycle conditions were used: initial denaturation at 95°C for 5 min followed by 15 cycles of denaturation at 95°C for 30 s, annealing at 55°C for 30 s and extension at 72°C for 30 s with a final extension of 10 min at 72°C. Amplifications were carried out in an Veriti™ 96-Well Thermocycler (Applied Biosystems).

**Quantitative PCR (qPCR).** qPCR was performed using the Bio-Rad^®^ CFX 96 instrument (Bio-Rad, Hercules, CA, USA).

***LPV3* nested qPCR.** PCR reactions were carried out in 20 μL reaction volumes with 2 μl of the outer *LPV3* PCR product (generated in the first step either from root DNA or mycelial DNA for the standard curve) as the template with 250 nM each of the forward and reverse *LPV3N* primers **(Table 2)**. The amplification was carried out using 1x Sensimix SYBR No-ROX (Bioline Ltd, London, UK). PCR cycling conditions for *LPV3N* were initial denaturation at 95°C for 10 min followed by 40 cycles, each consisting of denaturation at 95°C for 5 s, annealing at 60°C for 5 s, and primer extension at 72°C for 5 s.

***Actin* qPCR.** PCR reactions were carried out in 20 μL reaction volumes with 20 ng root sample DNA as template (or different concentrations of uninfected root DNA in case of standard curve preparation) with 250 nM each of the forward and reverse *Actin* primers **(Table 2)**. The amplification was carried out using 1x Sensimix SYBR No-ROX (Bioline Ltd.). PCR cycling conditions for *Actin* were initial denaturation at 95°C for 10 min followed by 40 cycles, each consisting of denaturation at 95°C for 15 s, annealing at 60°C for 15 s, and primer extension at 72°C for 15 s.

No template controls with water instead of the root DNA were included in the experiment. Three technical replicates were conducted for each sample. Melting curves were acquired at the end of the PCR run over the range of 65 to 95°C, increasing the temperature stepwise by 0.5°C every 5 s to confirm that individual qPCR signals corresponded to a single homogenous amplicon. The amplified products were visualized on a 2% agarose gel stained with GelRed (Biotium Inc) using the GelDoc™ EZ Gel Documentation System (Bio-Rad Laboratories, Hercules, CA, USA).

#### Re-isolation of *P*. *cinnamomi* from infected roots

Small root sections from 7-day-old infected roots were surface sterilized in ethanol, rinsed in sterile distilled water and plated onto NARPH *Phytophthora* selective medium [29] and incubated in darkness at 24°C. The plates were observed for typical rosette growth pattern of *P*. *cinnamomi*. Further, DNA was isolated from the mycelia as mentioned above and molecular confirmation was carried out by PCR amplification of *LPV3* [28].

### Statistical analysis

Linear regression analysis was performed on *Actin* and *LPV3* standard curves (Microsoft^®^ Excel). Root induction and transformation efficiency data were subjected to ANOVA and Tukey’s HSD analyses (RStudio^®^). Pathogen load determination involved Mann Whitney Wilcoxon Test (Statistics Online Computational Resource software package).

## Results

### Generation of composite avocado plants

#### Induction of adventitious rooting *ex vitro* in avocado using *A*. *rhizogenes* strains and rooting hormone application

**Fig 2:**
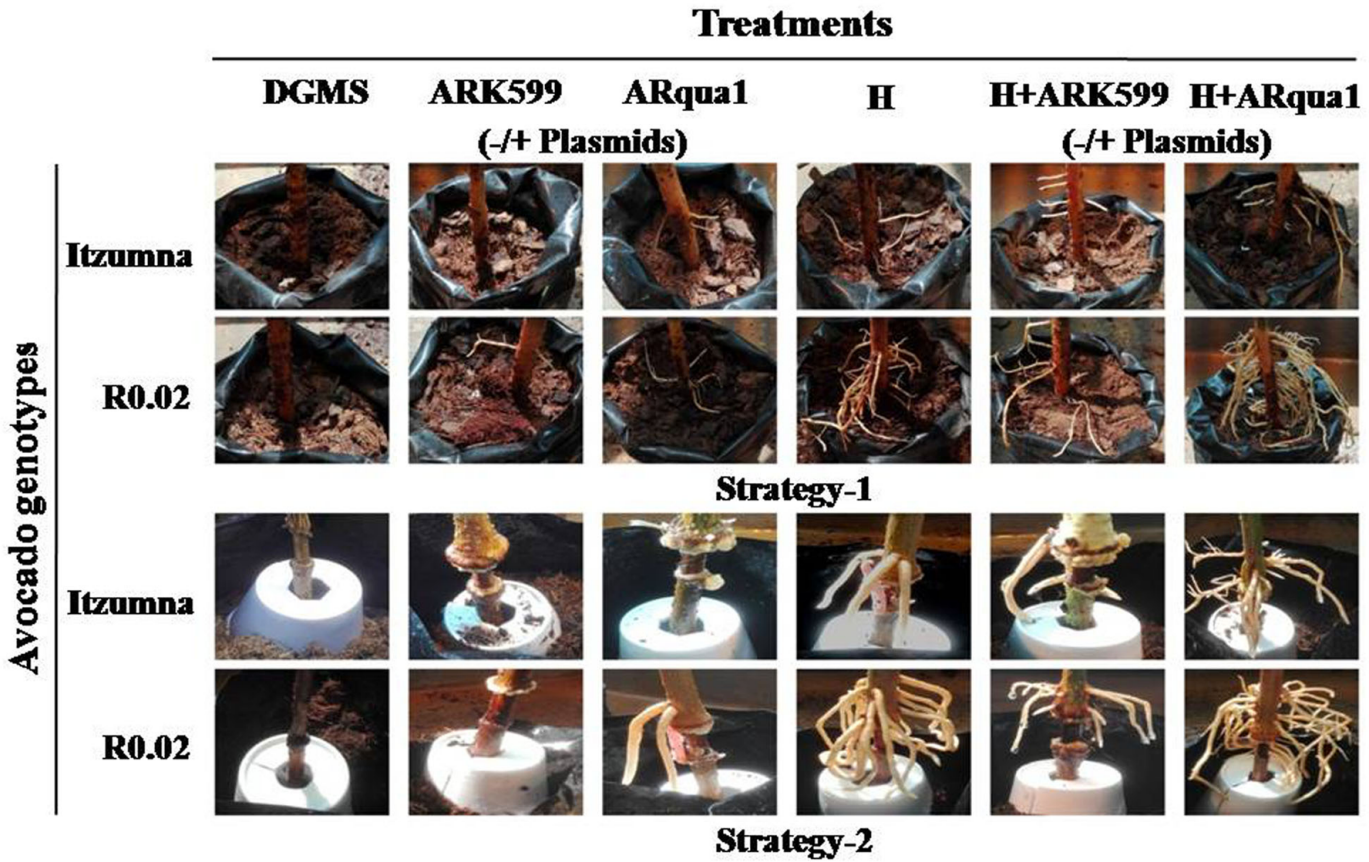
Representative images of the roots induced *ex vitro* in avocado plants by various treatments as detailed in Table 1.

Greenhouse-grown 8-week-old Itzamna and A0.74 avocado plants, etiolated and 20-week-old non-etiolated, were evaluated for their ability to induce the formation of adventitious roots by *ex vitro* strategies. Two *A*. *rhizogenes* strains- K599 and ARqua1, and rooting hormone treatments, individually and in combinations, were assessed **(Table 1)**. Root emergence was first observed in both avocado genotypes at 3 weeks post air-layering in hormone (H) and H+ARQ (-/+ plasmids)-treated plants. The H application was the best treatment for root induction. The *Agrobacterium* treatments on their own resulted in very few roots. Of the *Agrobacterium* treatments alone, ARqua1 was consistent in root induction in both avocado genotypes, across strategies. A marked difference in root vigour was observed between the two root-induction strategies, with Strategy-2 producing roots of greater thickness (**Fig 2**). A higher root branching was observed in H+ARQ (-/+ plasmids) treatment over the rest.

**Fig 3:**
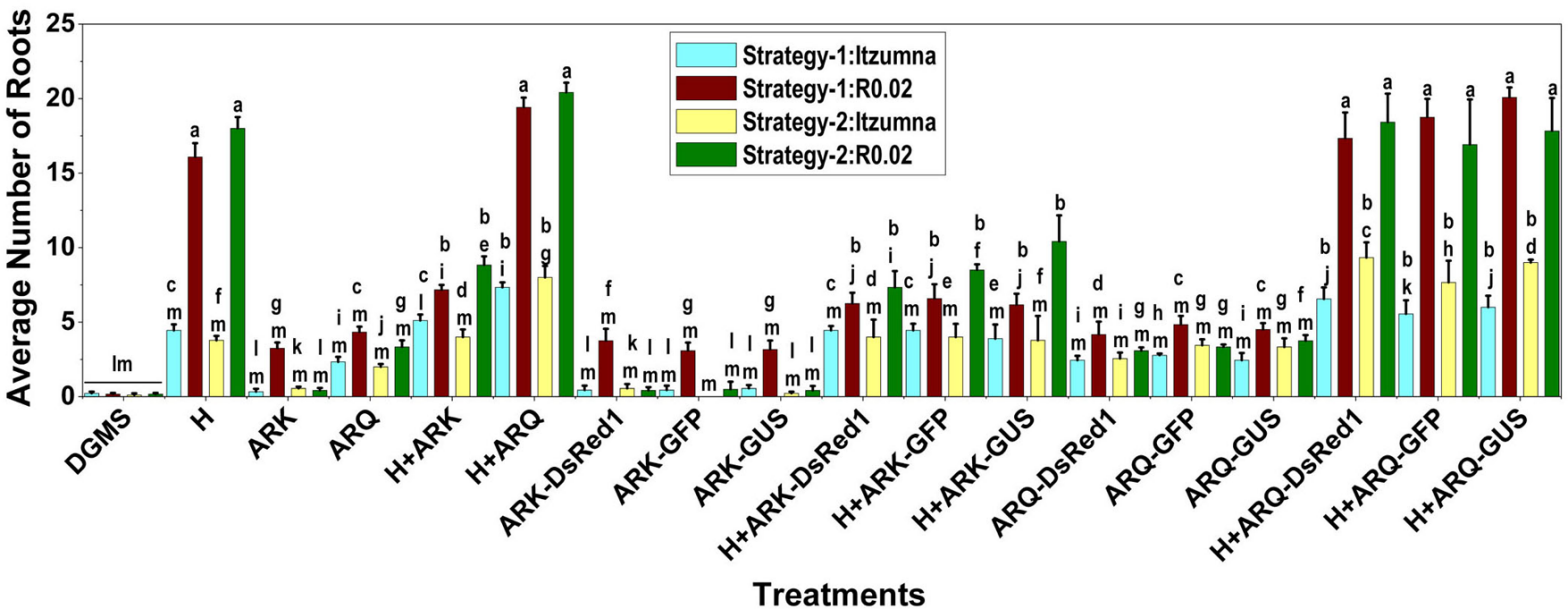
Average number of roots induced *ex vitro* in avocado plants by various treatments as detailed in Table 1. The study involved three biological replicates of three plants each per treatment. Bars indicate mean ± SE. Means designated with the same letter are not significantly different according to Tukey’s HSD test at P<0.05.

Considering the average number of roots induced per treatment, A0.74 showed >2-fold roots over Itzamna upon H treatment (**Fig 3**). The combination treatment of H+ARK599(/ARqua1) (-/+ plasmids) showed no significant difference in the root-induction capacity over the H-only treatment. Interestingly, only in A0.74, did the H+ARK treatment (-/+plasmids) show significantly fewer roots compared to H-only treatment, irrespective of the strategy used.

#### *A*. *rhizogenes*-mediated root transformation in avocado

**Fig 4:**
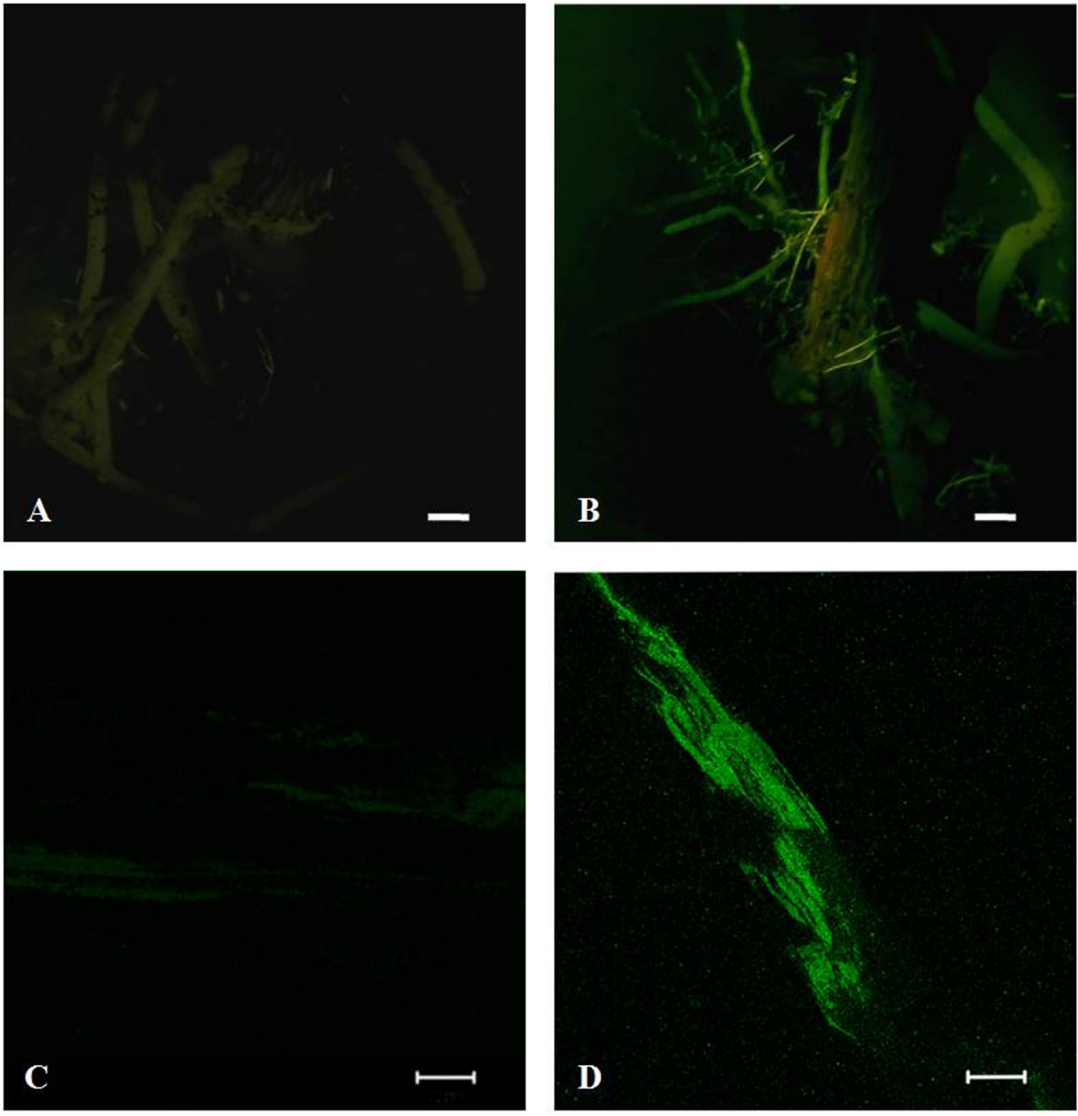
GFP fluorescence expression in roots of composite A0.74 avocado plants induced by Strategy-2. Root visualization using DFP-1^™^ Dual Fluorescent Protein Flashlight (NIGHTSEA, Bedford, MA, USA) (upper panel), Bar = 1 cm; roots under Zeiss LSM 510 META confocal laser scanning fluorescence microscope (Carl-Zeiss, Jena, Germany) (lower panel). **(A, C)**- H+ARQ and **(B, D)**- H+ARQ-GFP, Bar = 50 μm.

**Fig 5:**
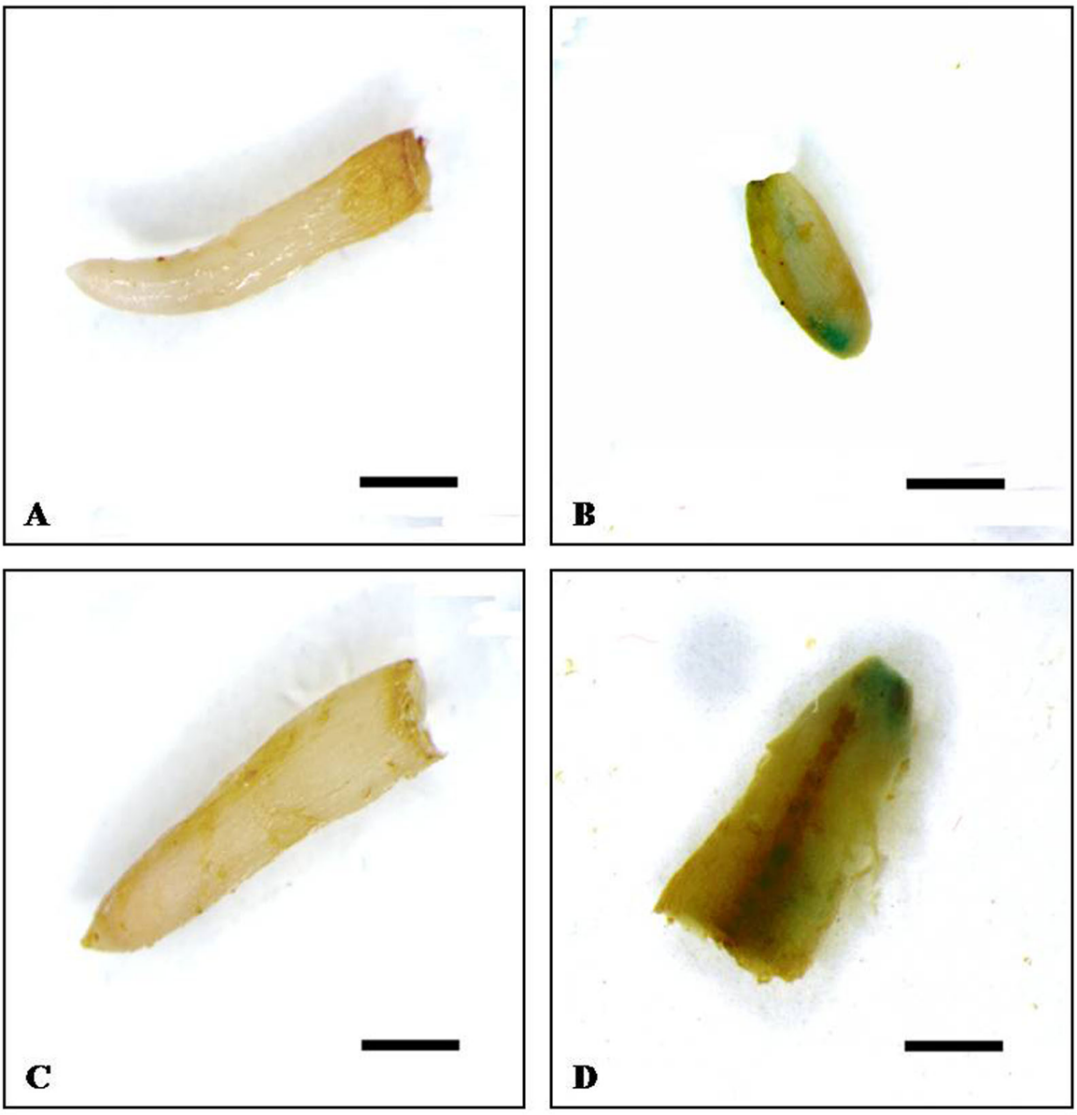
GUS activity staining in *ex vitro* roots induced in composite A0.74 avocado plants. The roots were visualized under SteREO Discovery.V12 stereomicroscope (Carl-Zeiss, Jena, Germany) fitted with AxioCam ICc5 (Carl-Zeiss, Jena, Germany). Upper panel represents the roots of Strategy-1 and the lower panel shows the roots of Strategy-2. **(A, C)**- H+ARQ controls and **(B, D)**- H+ARQ-GUS. Bar = 500 μm.

*A*. *rhizogenes* is known to induce adventitious roots, some of which are co-transformed with the gene-of-interest as well as roots lacking this gene [23]. The application of H enhances rooting capacity in avocado (personal communication, Dr. Stefan Kӧhne, Westfalia Technical Services, Tzaneen, Limpopo, South Africa) and is used commonly in commercial nurseries to generate clonal avocado plants. Hence, the *Agrobacterium*-only and in combination with H was employed with the intention of achieving higher root transformation efficiencies. Transformation events were monitored by using the binary vectors: pRedRootII, pBYR2e1-GFP and pBINUbiGUSint which express the fluorescent proteins- DsRed1, GFP and GUS enzyme, respectively which can be tracked by histochemical staining. Roots were assessed 6 weeks post air-layering of plants. Transformation efficiency per treatment/avocado genotype was defined as the percentage of experimental plants which resulted in at least one DsRED1/GFP/GUS expressing root. Only the ARqua1 (+plasmid) treatment of A0.74 resulted in root transformants, with H+ARqua1 transformed with pBINUbiGUSint being the most effective treatment, as the percentage of composite plants produced was ~17 and 25%, through strategies 1 and 2, respectively **(Table 3)**. The GFP expression was observed uniformly along the entire length of the roots, whereas, the GUS activity was restricted to the actively growing root tips (**Figs 4 and 5**). The weak background levels of green fluorescence observed in the control roots under the confocal microscope could easily be distinguished from the strong GFP fluorescence in roots transformed with pBYR2e1-GFP. None of the plants treated with the *Agrobacterium* containing pRedRootII resulted in any root transformants. The GUS and GFP expressing roots accounted for ≤4 and ~12%, respectively, of the total root population per treatment per avocado genotype. However, the average number of transgenic roots on the composite plants was <1 per plant in all treatments.

**Table 3:** Transformation efficiencies of the two strategies employed in the generation of *ex vitro* composite avocado plants.

Treatment	Strategy	Avocado genotype	% of composite plants	% of transformed roots	Average no. of transformed roots per plant
ARQ-GFP	2	A0.74	8.3 ± 0.3^b^	11.9 ± 1.7^a^	0.4 ± 0.4^ab^
H+ARQ-GUS	1		16.7 ± 0.3^ab^	0.9 ± 0.3^b^	0.2 ± 0.1^b^
	2		25 ± 0.6^a^	3.6 ± 1.1^ab^	0.8 ± 0.5^a^

**Strategy-1**: The 8-week-old etiolated seedlings were scarred six times with a sterile hacksaw blade at the base of the shoot on opposite surfaces. Strategy-2: One-inch-long incisions were made at the shoot base of 20-week-old plants grown under normal 16 h/8 h light/dark conditions with a sterile surgical blade to remove the cortical tissue. The scarred/wounded shoot surfaces were immediately subject to treatments as detailed in **Table 1**, using separate paint brushes. The treated shoot regions were covered with a 175 mL foam cup filled with sterile moist cocopeat. Plants were maintained under 16 h light/8 h dark in a phytotron at 25°C and the root induction was monitored regularly. In both approaches 3 biological replicates of 3 plants each were used. Data represents Means ± SE. Means designated with the same letter are not significantly different according to Tukey’s HSD test at P < 0.05.

### Molecular confirmation of root transformation in avocado

**Fig 6:**
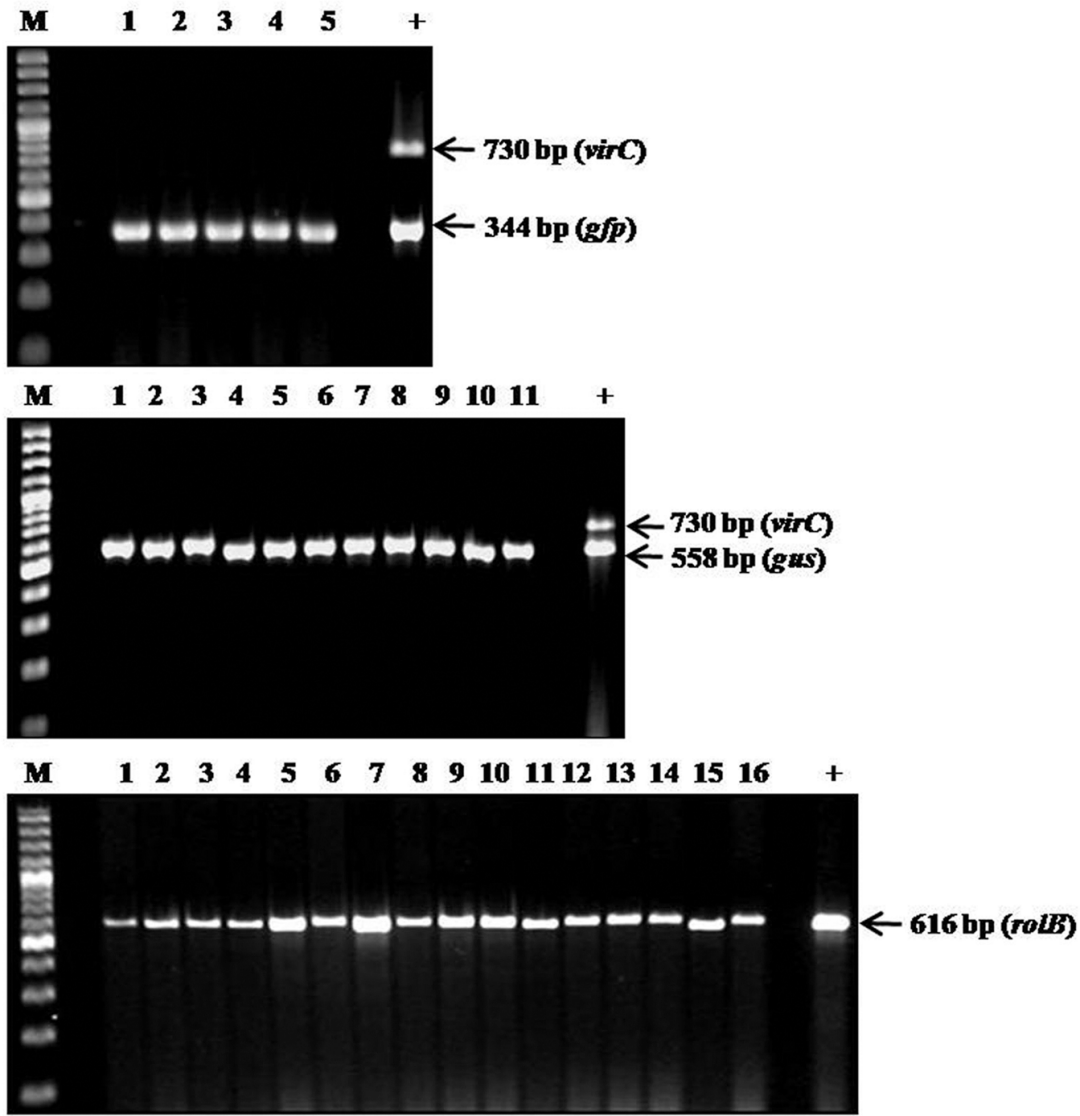
PCR confirmation of transgenic nature of the antibiotic-treated *ex vitro* roots induced in composite A0.74 avocado plants. The image shows agarose gel electrophoresis of PCR-amplified products of: **(A)** *gfp* and *virC*. Lanes 1–5 represent individual root samples expressing GFP from Strategy-2; **(B)** *gus* and *virC*. Lanes 1–2 represent individual root samples expressing GUS from Strategy-1 and Lanes 3–11 represent individual root samples expressing GUS from Strategy-2; **(C)** *rolB*. Lanes 1–2 represent individual root samples expressing GUS from Strategy-1, Lanes 3–11 represent individual root samples expressing GUS from Strategy-2 and Lanes 12–16 represent individual root samples expressing GFP from Strategy-2. M- GeneRuler 100 bp Plus DNA Ladder (Thermo Fischer Scientific) and + is the positive bacterial control.

**Fig 7:**
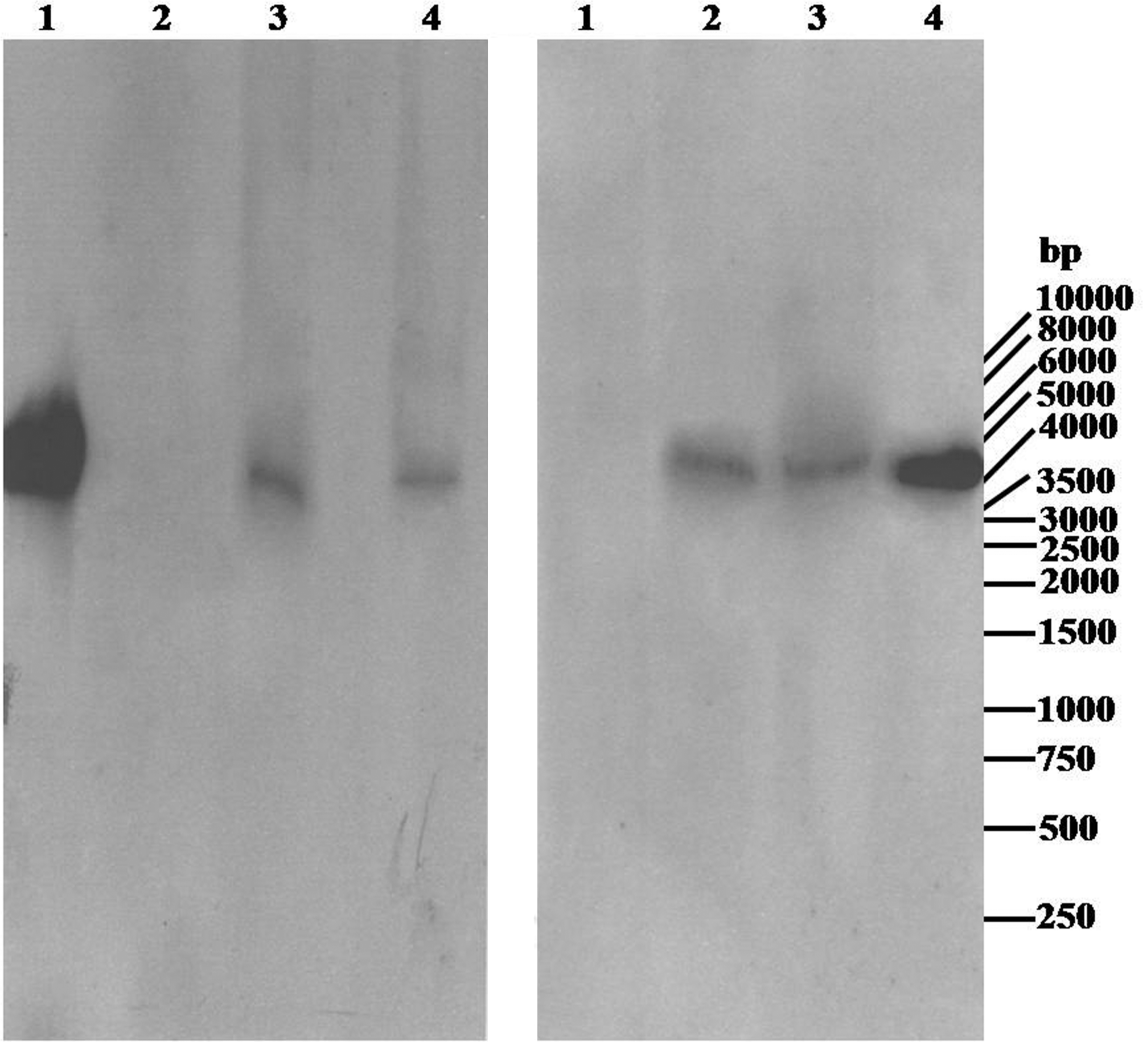
Southern blot analysis of transgenic avocado root DNA. *Hind*III digested DNA (5 μg each) from two independent GUS/GFP expressing composite A0.74 roots were separated on 0.8% agarose gel, transferred onto a positively charged nylon membrane and probed with *Hind*III restriction fragments of pBINUbiGUSint/pBYR2e1-GFP random-prime labelled with digoxigenin-dUTP. The chemiluinescent signals captured onto an X-ray film has been displayed. (**A)** GUS expressing composite A0.74 roots. Lanes: 1–500 pg of unlabelled probe fragment (hybridisation control), 2- *Hind*III digested untransformed A0.74 root DNA (negative control), 3- *Hind*III digested A0.74 root-1 DNA expressing GUS and 4- *Hind*III digested A0.74 root-2 DNA expressing GUS **(B)** GFP expressing composite A0.74 roots. Lanes: 1- *Hind*III digested untransformed A0.74 root DNA (negative control), 2- *Hind*III digested A0.74 root-1 DNA expressing GFP, 3- *Hind*III digested A0.74 root-2 DNA expressing GFP and 4–250 pg of unlabelled probe fragment (hybridisation control).

Root sections were cultured in antibiotic-containing media to eliminate the agrobacteria populating the transformed roots. Genomic DNA was isolated from these roots and PCR using appropriate gene-specific primers **(Table 2)** confirmed successful root transformation. The roots showing GFP fluorescence or GUS activity produced PCR products of their respective genes as expected. In addition, the same roots were also positive for the *rolB* gene indicating co-transformation of T-DNAs from Ri-plasmid. The lack of *virC* amplification in the same root DNA samples ruled out the presence of the screenable marker gene in the roots due to bacterial contamination and thus established the transgenic nature of the roots (**Fig 6**). Control wild-type plants tested negative for all of the above genes. Further, Southern blot analysis was performed to prove that the GUS and GFP expression observed in composite roots was due to stable integration and not transient expression of the T-DNAs carrying the respective genes. The *Hind*III restriction fragments of pBYR2e1-GFP and pBINUbiGUSint plasmids which contained the *gfp* and *gus* (*uidA-int*) genes, respectively, random-prime labelled with digoxigenin (DIG)-dUTP served as probes. These labelled *gfp* and *gus* gene probes were expected to identify 4438 bp and 4364 bp DNA fragments from the *Hind*III digested corresponding avocado root transformants, respectively. The Southern blot results involving two roots each expressing GFP and GUS confirmed the stable integration of the respective marker genes into the *A*. *rhizogenes* induced root genome (**Fig 7**). The wild-type A0.74 root DNA expectedly showed no reaction to the probes.

### Comparison of the infection of transgenic and wild-type avocado roots by *P*. *cinnamomi*

**Fig 8:**
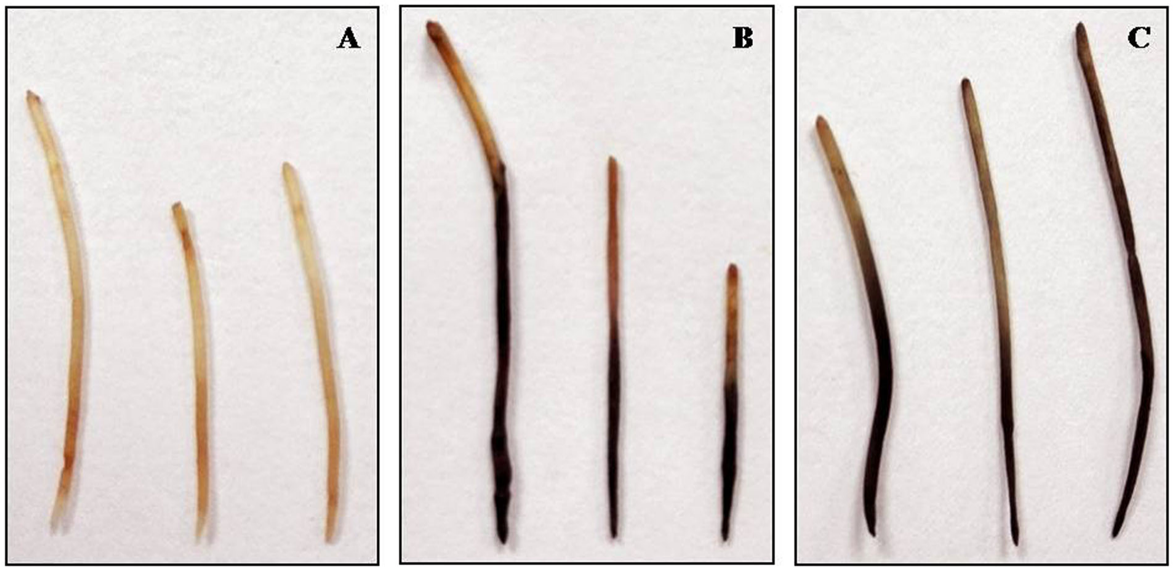
Root rot symptoms on A0.74 roots 7 days post *Phytophthora cinnamomi* infection. Roots were inoculated by submerging them in a petri plate containing 25 mL zoospore suspension at a concentration of 5×10^4^ zoospores/mL. **(A)** Mock-inoculated A0.74 roots submerged in sterile water (negative control); **(B)** Wild-type A0.74 roots inoculated with *P*. *cinnamomi* zoospores; and **(C)** Transgenic A0.74 roots expressing GFP inoculated with *P*. *cinnamomi* zoospores. The study involved three roots per treatment.

To determine if the transgenic roots from composite plants are amenable to *P*. *cinnamomi* infection the GFP-expressing roots and A0.74 wild-type control roots were infected with pathogen zoospores. Both transformed and wild-type A0.74 roots developed root rot symptoms typical of *P*. *cinnamomi* infection, with the mock-inoculated wild-type A0.74 roots remaining healthy **(Fig 8)**. Further, total genomic DNA was extracted from the above mentioned root samples which contained both plant and pathogen DNA, to determine the pathogen load by qPCR. The pathogen loads were very similar in both the transformed and wild-type A0.74 roots **(Table 4)**. In addition, pathogen re-isolation from the infected root sections and its molecular confirmation was successfully performed to prove Koch’s postulates.

**Table 4:** *Phytophthora cinnamomi* quantification in infected A0.74 avocado roots 7 days post inoculation.

Sample	Pathogen load (ng *P*. *cinnamomi* DNA/100 ng A0.74 root DNA)
A0.74 uninfected roots	0
A0.74 infected roots	10.002 ± 0.18
A0.74-GFP infected roots	9.897 ± 0.04

Pathogen load was determined from infected root tissues (three roots per treatment) by normalizing the *LPV3N* values with the corresponding *Actin* values for each individual sample. Data presented in the table are the means ± SE and were analysed with the Statistics Online Computational Resource software package using a Mann Whitney Wilcoxon Test (P < 0.001). No significant difference was observed in pathogen load between the infected A0.74 and A0.74-GFP roots.

## Discussion

Researchers rely on model organisms to understand the cellular and molecular aspects of life [30], as they have short generation times and are easily pliable to genetic manipulation. However, model systems do not always account for all the interactions that non-model plants encounter. Hence, to uncover specific responses encountered by non-model organisms in their niche an efficient transformation tool is a prerequisite. The developmental biology of the economically important fruit tree, avocado and its interaction with various abiotic and biotic factors is not well understood. A draft genome of the avocado has been sequenced and assembled but is not published. However, at least three genomes have been sequenced and are available for *P*. *cinnamomi* (JGI Genome Portal, USA), the major biotic factor affecting avocado production in countries where the pathogen is present. However, a high-throughput whole plant transformation tool for functional analysis of genes-of-interest remains a major limitation for advances in avocado research. Currently, the available *A*. *tumefaciens*-based plant transformation and regeneration protocols for avocado involves plant tissue culture necessitating specialized infrastructure and handling [9,11–14]. In addition, the development of stable transgenic plants is expensive, time consuming and not adapted for high-throughput functional screening of host and pathogen genes.

*A*. *rhizogenes* has been successfully employed in the composite plant generation of a number of woody plants such as poplar, coffee, grapevine and *Eucalyptus camadulensis* 31–34]; and has been the only available option in the transformation of recalcitrant trees such as black locust and larch [35,36]. Composite plant generation through *A*. *rhizogenes*-mediated transformation has enabled researchers to carry out the functional analysis of genes through gene overexpression, RNA interference-mediated gene silencing and promoter analysis [21,23,37–40]. Hence, the present research was undertaken to develop such a tool to transform avocado for down-stream application in functional research.

Initially, composite plant generation was attempted according to the *ex vitro* protocol described by [21], wherein, the *in vitro* regenerated shoots from avocado zygotic embryos, and apical shoot cuttings from various avocado genotypes were employed as explants. Less than 10% *in vitro* regenerated shoots treated with *A*. *rhizogenes* strains- K599 and ARqua1 resulted in root induction and no root co-transformation events were observed. Similar treatments of young apical shoot cuttings resulted only in the tumor-like growth with no root induction (S1 Fig). In a study involving the clonal propagation of Dutch Elms it was reported that wounded shoots treated with a combination of growth regulators and *A*. *rhizogenes/A*. *tumefaciens*-ORF-11 (ORF- open reading frame) significantly improved the rooting over the individual treatments [41]. In addition, the study also reported root transformation events. Thus, an improvisation of this strategy was employed in the present study. The term ‘*ex vitro* composite’ in this manuscript is used in a generic sense to indicate the tissue culture-free approaches in generating the adventitious roots in avocado and has nothing to do with the methodology of [21]. The effect of two different *A*. *rhizogenes* strains- K599 and ARqua1 transformed with screenable markers, individually and in combination with the rooting hormone, was assessed for their efficiency in rooting and transformation on etiolated and non-etiolated avocado plants (Itzamna and A0.74). Etiolated plants were considered in the study, as plants grown under dark or low light have been shown to root better not just in avocado but also in other plant systems [42,43]. The growth regulator application followed in the study was similar to that used in the commercial avocado nurseries at Westfalia Technical Services, Tzaneen, South Africa. In the present study, the application of the hormone significantly resulted in the best root induction, with the *Agrobacterium* treatments on their own resulting in very few roots. This is consistent with the fact that application of rooting hormone exogenously speeds-up the process of antioxidant enzyme synthesis, reduces the rooting time, and thus promotes root formation [44]. The combination treatment of H+ARK599/ARqua1 (-/+ plasmids) showed no significant difference in the root-induction capacity over the H-only treatment. This was unlike what was reported in the case of Dutch Elm [41] which could be due to the differences in the plant systems, the *Agrobacterium* strains and the nature and quantity of rooting hormones employed. ARqua1 is an agropine strain with *rol* genes A, B, C and D, and also auxin coding genes. Strain K599, cucumopine type on the other hand has only *rol* genes A, B and C [17,45]. This difference between the strains in addition to their differential interaction with the plant in the presence of the rooting hormone and the differences in age of the plants could explain the differential root vigour observed between the two strategies employed in our study. *A*. *rhizogenes* strains used in plant transformation have been shown to respond differentially to exogenous hormone treatments [46]. The roots induced in avocado did not show the typical hairy root phenotype. However, higher root branching observed in H+ARQ (-/+ plasmids) treatment over the rest could be due to the perturbations in the hormonal physiology, especially the auxin sensitivity of *A*. *rhizogenes*-induced roots [18]. Hyperbranching and plagiotrophy of the roots have been reported earlier from other plant systems transformed with *A*. *rhizogenes* [21,47]. The morphology of roots induced by *A*. *rhizogenes* have been reported to be variable depending upon the host:rhizobia combination. Both hairy roots (eg. *Casuarina gluaca*, *E*. *camadulensis*) and normal roots (eg. potato) have been observed in different plant systems [34,48,49]. The *rol* genes are known to induce and regulate root formation by interfering with the plant hormonal physiology [17,50]. The auxin levels are critical in rooting [51]. The interplay between the exogenously applied rooting hormone and strain K599 might have led to an imbalance in the auxin levels in A0.74 avocado rootsock leading to the observed lower number of roots compared to H-only treatment. Further, the known presence of additional ORFs on the Ri-plasmid T-DNA whose function is not well characterized could also influence the above phenotypes.

Numerous studies have reported *A*. *rhizogenes* K599 to be hyper-virulent and induce rooting in a wide range of dicotyledonous and monocotyledonous plants [17,45]. However, ARqua1 was the better of the two bacterial strains in root induction as well as transformation in the present study. ARqua1(+plasmid):A0.74 was the only successful combination that resulted in root transformants, with H+ARqua1(+pBINUbiGUSint) being the most effective treatment. The host:*Agrobacterium* interaction is complex and is not fully understood. Certain combinations of host genotypes and bacterial strains have been reported to be more efficient in rooting and transformation in other plant systems as well [49]. In addition, interactions between the genetic background of the *Agrobacterium*, binary vectors, acetosyringone concentrations and pH have been shown to have an impact on avocado transformation efficiencies [12]. Highest transformation efficiencies were achieved in A0.74 upon H+ARqua1(+pBINUbiGUSint) infection. Both transgenic and non-transformed adventitious roots have been reported to be induced by *A*. *rhizogenes* in recalcitrant woody species [41,52]. In the present study, the percentage of transformed roots was low, varying from ~1–12% of the total root population for the different treatments and the average number of transgenic roots on the composite plants was <1 per plant in all treatments. In an earlier study which assessed the efficiency of composite plant production from 14 plant species, the generation time for the transgenic roots was found to be dependent on the host plant which varied from 24 to 67 days for *Nicotiana tabacum* and *Petunia hybrida*, respectively. Most plant species produced transgenic roots with an average time of about 6 weeks consistent with our results [21]. The transformation efficiencies varied from 56% in *Medicago truncatula* to 100% in *N*. *tabacum* and *P*. *hybrida*, which was higher than that reported for avocado in the present study. The percentage of the transgenic roots varied from 19% (*N*. *benthamiana*) to 58% (*N*. *tabacum*) and the average number of transgenic roots on *ex vitro* composite plants ranged from 1 (*M*. *truncatula*) to ~10 per explant (*N*. *tabacum*). Most of the plants used in the study showed four or less roots per explant. In another study carried out in potato using four different cultivars and eight different *A*. *rhizogenes* strains the transformation efficiencies were found to be dependent on the cultivar:strain combinations with a range of 0–100% [49]. The cucumopine and mannopine strains were found to be less efficient than the agropine strains in inducing transgenic roots. This is in line with the present study where the agropine strain was the only successful avocado transformer. However, further multiple strains of the different opine producers need to be tested against different avocado genotypes before any definite conclusions can be drawn.

In woody tree species such as poplar, coffee and eucalyptus composite plants were generated *in vitro* using different explants. Stems of different poplar clones infected with *A*. *rhizogenes* R1000 resulted in transformation efficiencies of 17–92% [31]. Coffee embryos showed transformation efficiencies of 70% with 35% roots being transgenic [32]. In eucalyptus, the transformation efficiencies of 36% and 4.1% with ~1 and ~0.5 roots per plant were recorded with seedling explants and *in vitro* grown plantlets, respectively [34]. The lower transformation efficiencies in the present study compared to the above studies could be due to the differences in age and nature of the explants in addition to the bacterial strains and the infection conditions. The present study successfully explored the possibility of generation of composite plants *ex vitro*, which has been achieved. However, an extensive large scale follow-up study employing more avocado genotypes, rhizobial strains, infection conditions need to be optimized to achieve higher transformation efficiencies to be able to carry out high-throughput functional gene characterization studies.

*A*. *rhizogenes* transformed with binary vectors- pRedRootII, pBYR2e1-GFP and pBINUbiGUSint which express the fluorescent proteins- DsRed1, GFP and GUS enzyme, respectively, were used to monitor the root transformation. Unlike in other plant tissues such as leaves and stems [53], the low auto-fluorescence observed in the roots did not interfere with the identification of the transgenic roots, which displayed strong GFP expression. The restricted GUS activity spots to the actively growing root tips and not the whole root could be due to the poor diffusion of the substrate into the root interior. Though DsRed1 expressing vectors have been used successfully in avocado [13], no transformants were observed here which could have been due to insertion of the T-DNA harbouring *dsred1* in a transcriptionally inactive region of the chromatin (few randomly chosen roots from pRedRootII-treated plants however turned negative for *dsred1*) or the *Arabidopsis* Pubq10 promoter may not be functional in avocado. However, the sunflower polyubiquitin promoter has been successfully used in avocado transformation [14].

The GFP and GUS expression in the roots could not have been of bacterial origin due to leaky expression, as their encoding genes contain introns which cannot be processed by prokaryotes. Still, molecular confirmation of the transgenic nature of the roots was achieved by antibiotic treatment of the roots followed by successful amplification of the screenable marker (*gfp* and *gus*) and *rolB* genes, and the lack of amplification of *virC*. The *virC* gene is one of the multiple *vir* or virulence genes present on the Ri or Ti plasmids which encode enzymes aiding in the transfer and integration of the T-DNA into the plant cells without itself getting integrated [19]. In *A*. *rhizogenes*-induced hairy roots *virC* or *virD* genes have been used earlier to confirm the transgenic nature of the roots by establishing the absence of *Agrobacterium* contamination in the hairy roots [54,55]. Further, Southern blot analysis results involving two roots each expressing GFP and GUS confirmed the stable integration of the respective marker genes into the *A*. *rhizogenes*-induced root genome.

The ultimate aim of generating composite avocado plants, in our case, is to study the underlying molecular interaction between the transgenic plant and the root pathogen *P*. *cinnamomi*. It is critical to ensure that the transgenic roots are similar to wild-type roots at both the morphological and physiological levels before taking up further interaction studies, as *A*. *rhizogenes*-induced roots have been shown to have altered phenotypes [49]. Only GFP-expressing transgenic roots were employed in the pathogen infection studies as the GUS-transgenic roots were subjected to destructive histochemical analysis. Though the transgenic avocado roots did show some hyper-branching their response to the infection by *P*. *cinnamomi* was very similar to the non-transformed roots of the same avocado genotype. *A*. *rhizogenes*-derived roots from various host plants have earlier been used in understanding their interaction with bacteria, fungi, oomycetes, nematodes and parasitic plants 21, 55–61]. Altered physiology of *M*. *truncatula* hairy roots was shown to have no impact on its interaction with the fungus *Glomus intraradices* [62]. Further, hormonal profiling of the transgenic and wild-type avocado roots need to be carried out in order to detect any differences in their physiology, and further determine the impact of such differences on the host-pathogen interaction at the molecular level.

## Conclusions

For the first time the research presented in this study has provided a proof-of-concept composite plant system for avocado which is relatively easy, quick and cost-effective compared to the *in vitro* transformation approaches. Strategy-2 involving A0.74:ARqua1 combination was found to be the best approach in producing composite avocado plants. Further, studies need to be carried out to assess its adaptability in the generation of composite plants in multiple avocado genotypes for its deployment in high-throughput genetic analysis to study not just the biotic and abiotic factors afflicting avocado, but to also investigate the root developmental biology.

## Supporting information

S1 Fig Representative image showing the composite plant generation attempted in avocado according to the *ex vitro* protocol described by [21]. (A) Root induction observed when *in vitro* regenerated shoots from avocado zygotic embryos used as explant. (B) Tumor-like growth with no root induction observed with young apical shoot cuttings as explants.
(TIF)

## References

[1] FAOSTAT. http://www.fao.org/faostat/en/?#data/QC. 2014.

[2] Mogala M. A profile of the South African avocado market value chain. Dep Agric For Fish Repub South Africa. 2015; 1–52. doi: 10.1017/CBO9781107415324.004

[3] Zentmyer G. *Phytophthora cinnamomi* and the diseases it causes Amer. Phytopathol. Soc. Monogr. St. Paul, Minn: American Phytopathological Society; 1980.

[4] Acosta-Muniz CH, Escobar-Tovar L, Valdes-Rodriguez S, Fernandez-Pavia S, Arias-Saucedo LJ, de la Cruz Espindola Barquera M, et al Identification of avocado (*Persea americana*) root proteins induced by infection with the oomycete *Phytophthora cinnamomi* using a proteomic approach. Physiol Plant. 2012;144: 59–72. doi: 10.1111/j.1399-3054.2011.01522.x 21916897 10.1111/j.1399-3054.2011.01522.x

[5] Pegg KG, Coates LM, Korsten L, Harding RM. Foliage, fruit and soilborne diseases In: Whiley AW, Schaffer B, Wolstenholme BN, editors. Avocado: botany, production and uses. Wallingford: CABI Publishing, England; 2002 pp. 432–456.

[6] Hardy GESJ, Barrett S, Shearer BL. The future of phosphite as a fungicide to control the soilborne plant pathogen *Phytophthora cinnamomi* in natural ecosystems. Australas Plant Pathol. 2001;30: 133–139. doi: 10.1071/AP01012

[7] Pérez-Jiménez RM. Significant avocado diseases caused by fungi and oomycetes. Eur J Plant Sci Biotechnol. 2008;2: 1–24.

[8] Darvas J, Bezuidenhout J. Control of Phytophthora root rot of avocados by trunk injection. South African Avocado Growers’ Association Yearbook. 1987 pp. 91–93.

[9] Raharjo SHT, Witjaksono, Gomez-Lim MA, Padilla G, Litz RE. Recovery of avocado (*Persea americana* Mill.) plants transformed with the antifungal plant defensin gene PDF1.2. Vitr Cell Dev Biol—Plant. 2008;44: 254 doi: 10.1007/s11627-008-9117-2

[10] Coffey M. Phytophthora root rot of avocado In: Kumar J, Chaube H, US S, Mukhopadhyay A, editors. Plant Diseases of International Importance Vol III Diseases of Fruit Crops. New Jersey, USA: Prentice Hall, Englewood Cliffs; 1992 pp. 423–444.

[11] Cruz-Hernández A, Litz WRE, Lim MG. *Agrobacterium tumefaciens*—mediated transformation of embryogenic avocado cultures and regeneration of somatic embryos. Plant Cell Rep. 1998;17: 497–503. doi: 10.1007/s002990050431 10.1007/s002990050431 30736625

[12] Ahmed MF, Kantharajah AS, Holford P. Genetic transformation studies on avocado cultivar “Hass” (*Persea americana*). Am J Plant Sci. 2012;3: 1225–1231.

[13] Palomo-Ríos E, Cerezo S, Mercado JA, Pliego-Alfaro F. *Agrobacterium*-mediated transformation of avocado (*Persea americana* Mill.) somatic embryos with fluorescent marker genes and optimization of transgenic plant recovery. Plant Cell Tissue Organ Cult. Springer Netherlands; 2017;128: 447–455. doi: 10.1007/s11240-016-1122-2

[14] Chaparro-Pulido CA, Montiel MM, Palomo-Ríos E, Mercado JA, Pliego-Alfaro F. Development of an efficient transient transformation protocol for avocado (*Persea americana* Mill.) embryogenic callus. In Vitro Cell Dev Biol—Plant. 2014;50: 292–298. doi: 10.1007/s11627-013-9564-2

[15] Taylor NJ, Fauquet CM. Microparticle bombardment as a tool in plant science and agricultural biotechnology. DNA Cell Biol. 2002;21: 963–977. doi: 10.1089/104454902762053891 12573053 10.1089/104454902762053891

[16] Riker A, Banfield W, Wright W, Keitt G, Sagen H. Studies on infectious hairy root of nursery-apple tree. J Agric Res. 1930;41: 507–540.

[17] Veena V, Taylor CG. *Agrobacterium* rhizogenes: Recent developments and promising applications. In Vitro Cell Dev Biol—Plant. 2007;43: 383–403. doi: 10.1007/s11627-007-9096-8

[18] Christey MC. Use of Ri-mediated transformation for production of transgenic plants. In Vitro Cell Dev Biol—Plant. 2001;37: 687–700. doi: 10.1007/s11627-001-0120-0

[19] Häggman H, Aronen T. Agrobacterium rhizogenes for rooting recalcitrant woody plants In: Jain SM, Minocha SC, editors. Molecular biology of woody plants: Volume 2. Dordrecht: Springer Netherlands; 2000 pp. 47–78. doi: 10.1007/978-94-017-2313-8_3

[20] Hansen J, Jørgensen JE, Stougaard J, Marcker KA. Hairy roots—a short cut to transgenic root nodules. Plant Cell Rep. 1989;8: 12–15. doi: 10.1007/BF00735768 24232586 10.1007/BF00735768

[21] Collier R, Fuchs B, Walter N, Lutke WK, Taylor CG. *Ex vitro* composite plants: An inexpensive, rapid method for root biology. Plant J. 2005;43: 449–457. doi: 10.1111/j.1365-313X.2005.02454.x 16045479 10.1111/j.1365-313X.2005.02454.x

[22] Kereszt A, Li D, Indrasumunar A, Nguyen CDT, Nontachaiyapoom S, Kinkema M, et al *Agrobacterium rhizogenes*-mediated transformation of soybean to study root biology. Nat Protoc. Nature Publishing Group; 2007;2: 948–952. Available: doi: 10.1038/nprot.2007.141 17446894 10.1038/nprot.2007.141

[23] Limpens E, Ramos J, Franken C, Raz V, Compaan B, Franssen H, et al RNA interference in *Agrobacterium rhizogenes*-transformed roots of Arabidopsis and *Medicago truncatula*. J Exp Bot. 2004;55: 983–992. doi: 10.1093/jxb/erh122 15073217 10.1093/jxb/erh122

[24] Vitha S, Beneš K, Phillips JP, Gartland KMA. Histochemical GUS Analysis In: Gartland KMA, Davey MR, editors. Agrobacterium Protocols. Totowa, NJ: Springer New York; 1995 pp. 185–193. doi: 10.1385/0-89603-302-3:185

[25] Brunner I. Molecular identification of fine roots of trees from the Alps: reliable and fast DNA extraction and PCR–RFLP analyses of plastid DNA. 2001; 2079–2087. Available: http://www3.interscience.wiley.com/cgi-bin/fulltext/118987698/PDFSTART 11555251 10.1046/j.1365-294x.2001.01325.x

[26] Wagenknecht M, Meinhardt F. Copy number determination, expression analysis of genes potentially involved in replication, and stability assays of pAL1—the linear megaplasmid of *Arthrobacter nitroguajacolicus* Ru61a. Microbiol Res. 2011;166: 13 doi: 10.1016/j.micres.2009.12.005 20116226 10.1016/j.micres.2009.12.005

[27] Engelbrecht J, Duong TA, van den Berg N. Development of a nested quantitative real-time PCR for detecting *Phytophthora cinnamomi* in *Persea americana* rootstocks. Plant Dis. 2013;97: 1012–1017. doi: 10.1094/PDIS-11-12-1007-RE 10.1094/PDIS-11-12-1007-RE 30722481

[28] Kong P, Hong CX, Richardson PA. Rapid detection of *Phytophthora cinnamomi* using PCR with primers derived from the *Lpv* putative storage protein genes. Plant Pathol. 2003;52: 681–693. doi: 10.1111/j.1365-3059.2003.00935.x

[29] Hüberli D, Tommerup IC, Hardy GESJ. False-negative isolations or absence of lesions may cause mis-diagnosis of diseased plants infected with *Phytophthora cinnamomi*. Australas Plant Pathol. 2000;29: 164 doi: 10.1071/AP00029

[30] Armengaud J, Trapp J, Pible O, Geffard O, Chaumot A, Hartmann EM. Non-model organisms, a species endangered by proteogenomics. J Proteomics. Elsevier B.V.; 2014;105: 5–18. doi: 10.1016/j.jprot.2014.01.007 24440519 10.1016/j.jprot.2014.01.007

[31] Han K, Gordon MP, Strauss SH. High-frequency transformation of cottonwoods (genus Populus) by *Agrobacterium rhizogenes*. Can J For Res. 1997;27: 464–470. doi: 10.1139/cjfr-27-4-464

[32] Alpizar E, Dechamp E, Espeout S, Royer M, Lecouls AC, Nicole M, et al Efficient production of *Agrobacterium rhizogenes*-transformed roots and composite plants for studying gene expression in coffee roots. Plant Cell Rep. 2006;25: 959–967. doi: 10.1007/s00299-006-0159-9 16596429 10.1007/s00299-006-0159-9

[33] Guellec V, David C, Branchard M, Temp J. *Agrobacterium rhizogenes* mediated transformation of grapevine (*Vitis vinifera* L.). Plant Cell, Tissue Organ Cult. 1990; 211–215. doi: 10.1007/BF00041883

[34] Balasubramanian A, Venkatachalam R, Selvakesavan KR, Mary A, Gherbi H, Svistoonoff S, et al Optimisation of methods for *Agrobacterium rhizogenes* mediated generation of composite plants in *Eucalyptus camaldulensis*. BMC Proc. 2011;5: O45 doi: 10.1186/1753-6561-5-S7-O45

[35] Han K-H, Keathley DE, Davis JM, Gordon MP. Regeneration of a transgenic woody legume (*Robinia pseudoacacia* L., black locust) and morphological alterations induced by *Agrobacterium rhizogenes*-mediated transformation. Plant Sci. 1993;88: 149–157. http://dx.doi.org/10.1016/0168-9452(93)90086-F

[36] Shin D-I, Podila GK, Huang Y, Karnosky DF. Transgenic larch expressing genes for herbicide and insect resistance. Can J For Res. NRC Research Press; 1994;24: 2059–2067. doi: 10.1139/x94-264

[37] Gonzalez-Rizzo S, Crespi M, Frugier F. The *Medicago truncatul*a CRE1 cytokinin receptor regulates lateral root development and early symbiotic interaction with *Sinorhizobium meliloti*. Plant Cell Online. 2006;18: 2680–2693. doi: 10.1105/tpc.106.043778 17028204 10.1105/tpc.106.043778 PMC1626621

[38] Gherbi H, Nambiar-Veetil M, Zhong C, Félix J, Autran D, Girardin R, et al Post-transcriptional gene silencing in the root system of the actinorhizal tree *Allocasuarina verticillata*. Mol Plant-Microbe Interact. 2008;21: 518–524. doi: 10.1094/MPMI-21-5-0518 18393611 10.1094/MPMI-21-5-0518

[39] Dalton DA, Boniface C, Turner Z, Lindahl A, Kim HJ, Jelinek L, et al Physiological roles of glutathione *S*-transferases in soybean root nodules. Plant Physiol. 2009;150: 521–530. doi: 10.1104/pp.109.136630 19279195 10.1104/pp.109.136630 PMC2675717

[40] Schaarschmidt S, Gresshoff PM, Hause B. Analyzing the soybean transcriptome during autoregulation of mycorrhization identifies the transcription factors GmNF-YA1a/b as positive regulators of arbuscular mycorrhization. Genome Biol. BioMed Central Ltd; 2013;14: R62 doi: 10.1186/gb-2013-14-6-r62 23777981 10.1186/gb-2013-14-6-r62 PMC3706930

[41] Rinallo C, Mittempergher L, Frugis G, Mariotti D. Clonal propagation in the genus Ulmus: Improvement of rooting ability by *Agrobacterium rhizogenes* T-DNA genes. J Hortic Sci Biotechnol. Taylor & Francis; 1999;74: 502–506. doi: 10.1080/14620316.1999.11511143

[42] Frolich EF, Platt RG. Use of the etiolation technique in rooting avocado cuttings. Society. 1971; 97–109.

[43] Pacholczak A, Szydło W, Łukaszewska A. The effect of etiolation and shading of stock plants on rhizogenesis in stem cuttings of *Cotinus coggygria*. Acta Physiol Plant. 2005;27: 417–428. doi: 10.1007/s11738-005-0046-y

[44] Zhang W, Fan J, Tan Q, Zhao M, Zhou T, Cao F. The effects of exogenous hormones on rooting process and the activities of key enzymes of *Malus hupehensis* stem cuttings. PLoS One. 2017;12: 1–13. doi: 10.1371/journal.pone.0172320 28231330 10.1371/journal.pone.0172320 PMC5322878

[45] Xiang T, Wang S, Wu P, Li Y, Zhang T, Wu D, et al Cucumopine type *Agrobacterium rhizogenes* K599 (NCPPB2659) T-DNA-mediated plant transformation and its application. Bangladesh J Bot. 2016;45: 935–945.

[46] Sauerwein M, Wink M, Shimomura K. Influence of light and phytohormones on alkaloid production in transformed root cultures of *Hyoscyamus albus*. J Plant Physiol. 1992;140: 147–152. http://dx.doi.org/10.1016/S0176-1617(11)80925-9

[47] Otani M, Shimada T, Kamada H, Teruya H, Mii M. Fertile transgenic plants of *Ipomoea trichocarpa* Ell. induced by different strains of *Agrobacterium rhizogenes*. Plant Sci. 1996;116: 169–175. doi: 10.1016/0168-9452(96)04394-4

[48] Diouf D, Gherbi H, Prin Y, Franche C, Duhoux E, Bogusz D. Hairy root nodulation of *Casuarina glauca*: a system for the study of symbiotic gene expression in an actinorhizal tree. Mol Plant Microbe Interact. 1995;8: 532–537. Available: http://www.ncbi.nlm.nih.gov/entrez/query.fcgi?cmd=Retrieve&db=PubMed&dopt=Citation&list_uids=8589409 8589409 10.1094/mpmi-8-0532

[49] Horn P, Santala J, Nielsen SL, Hühns M, Broer I, Valkonen JPT. Composite potato plants with transgenic roots on non-transgenic shoots: a model system for studying gene silencing in roots. Plant Cell Rep. 2014;33: 1977–1992. doi: 10.1007/s00299-014-1672-x 25182479 10.1007/s00299-014-1672-x

[50] Nilsson O, Olsson O. Getting to the root: The role of the *Agrobacterium rhizogenes rol* genes in the formation of hairy roots. Physiol Plant. 1997;100: 463–473. doi: 10.1034/j.1399-3054.1997.1000307.x

[51] Overvoorde P, Fukaki H, Beeckman T. Auxin control of root development. Cold Spring Harb Perspect Biol. 2010;2: 1–16. doi: 10.1101/cshperspect.a001537 20516130 10.1101/cshperspect.a001537 PMC2869515

[52] Falasca G, Reverberi M, Lauri P, Caboni E, De Stradis A, Altamura MM. How *Agrobacterium rhizogenes* triggers *de novo* root formation in a recalcitrant woody plant: An integrated histological, ultrastructural and molecular analysis. New Phytol. 2000;145: 77–93. doi: 10.1046/j.1469-8137.2000.00558.x

[53] Molinier J, Himber C, Hahne G. Use of green fluorescent protein for detection of transformed shoots and homozygous offspring. Plant Cell Rep. 2000;19: 219–223. doi: 10.1007/s002990050002 10.1007/s002990050002 30754898

[54] Kumar V, Sharma A, Prasad BCN, Gururaj HB, Ravishankar GA. *Agrobacterium rhizogenes* mediated genetic transformation resulting in hairy root formation is enhanced by ultrasonication and acetosyringone treatment. Electron J Biotechnol. 2006;9: 1–9. doi: 10.2225/vol9-issue4-fulltext-4

[55] Ishida JK, Yoshida S, Ito M, Namba S, Shirasu K. *Agrobacterium rhizogenes*-mediated transformation of the parasitic plant *Phtheirospermum japonicum*. PLoS One. 2011;6: 1–8. doi: 10.1371/journal.pone.0025802 21991355 10.1371/journal.pone.0025802 PMC3185032

[56] Quandt HJ, Puehler A, Broer I. Transgenic root nodules of *Vicia hirsuta*: a fast and efficient system for the study of gene expression in indeterminate-type nodules. Mol Plant Microbe Interact. 1993;6: 699–703. doi: 10.1094/MPMI-6-699

[57] Kifle S, Shao M, Jung C, Cai D. An improved transformation protocol for studying gene expression in hairy roots of sugar beet (*Beta vulgaris* L.). Plant Cell Rep. 1999;18: 514–519. doi: 10.1007/s002990050614

[58] Narayanan RA, Atz R, Denny R, Young ND, Somers DA. Expression of soybean cyst nematode resistance in transgenic hairy roots of soybean. Contribution no. 991130114 from the Minnesota Agric. Exp. Stn. This work was supported in part by USDA/95-37300-1593. Crop Sci. Madison, WI: Crop Science Society of America; 1999;39: 1680–1686. doi: 10.2135/cropsci1999.3961680x

[59] Noorda-Nguyen K, Mccafferty H, Aoki A, Nishijima W, Zhu YJ. *Agrobacterium rhizogenes* generates transgenic hairy roots in *Carica papaya* L.: A new approach for studying and improving resistance to the root-rot pathogen, *Phytophthora palmivora*. Transgenic Plant J; 2010;4: 94–96.

[60] Ilina EL, Logachov AA, Laplaze L, Demchenko NP, Pawlowski K, Demchenko KN. Composite *Cucurbita pepo* plants with transgenic roots as a tool to study root development. Ann Bot. 2012;110: 479–489. doi: 10.1093/aob/mcs086 22553131 10.1093/aob/mcs086 PMC3394650

[61] Mellor KE, Hoffman AM, Timko MP. Use of *ex vitro* composite plants to study the interaction of cowpea (*Vigna unguiculata* L.) with the root parasitic angiosperm *Striga gesnerioides*. Plant Methods; 2012;8: 22 doi: 10.1186/1746-4811-8-22 22741546 10.1186/1746-4811-8-22 PMC3441300

[62] Mrosk C, Forner S, Hause G, Küster H, Kopka J, Hause B. Composite *Medicago truncatula* plants harbouring *Agrobacterium rhizogenes*-transformed roots reveal normal mycorrhization by *Glomus intraradices*. J Exp Bot. 2009;60: 3797–3807. doi: 10.1093/jxb/erp220 19574251 10.1093/jxb/erp220 PMC2736893

