# Supplementary figures and images for: Generation of composite Persea americana (Mill.) (avocado) plants: A proof-of-concept-study
Source: PLoS One. 2017 Oct 20;12(10):e0185896. doi: 10.1371/journal.pone.0185896 (PMC5650140; doi:10.1371/journal.pone.0185896)

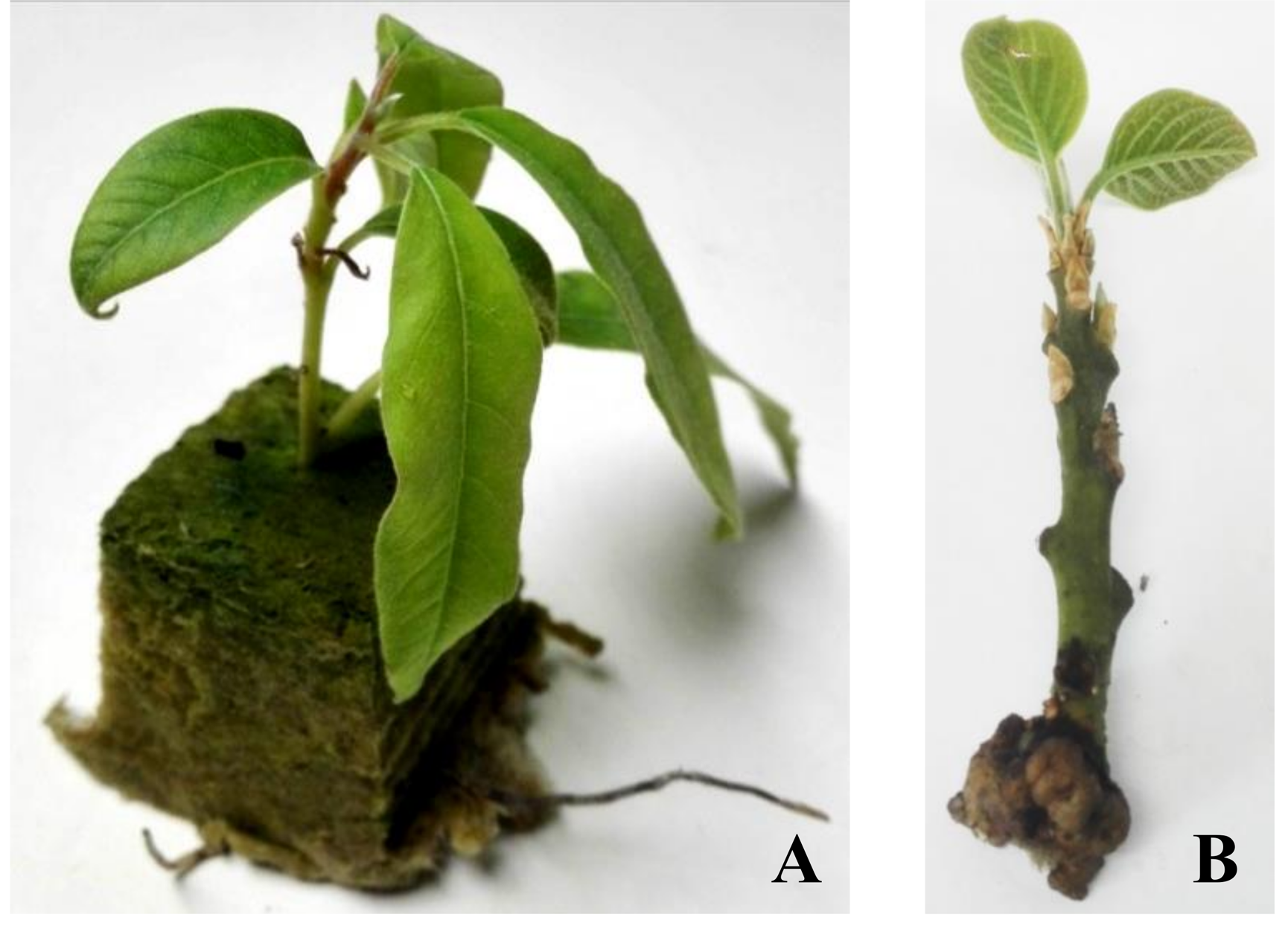

Supplement: S1 Fig — (A) Root induction observed when in vitro regenerated shoots from avocado zygotic embryos used as explant. (B) Tumor-like growth with no root induction observed with young apical shoot cuttings as explants. (TIF) [file pone.0185896.s001.tif]
